# A Novel *CD3G* Mutation in a Taiwanese Patient With Normal T Regulatory Function Presenting With the CVID Phenotype Free of Autoimmunity—Analysis of all Genotypes and Phenotypes

**DOI:** 10.3389/fimmu.2019.02833

**Published:** 2019-12-19

**Authors:** Wen-I Lee, Wen-Lang Fan, Chun-Hao Lu, Shih-Hsiang Chen, Ming-Ling Kuo, Syh-Jae Lin, Weng-Sheng Tsai, Tang-Her Jaing, Li-Chen Chen, Kuo-Wei Yeh, Tsung-Chieh Yao, Jing-Long Huang

**Affiliations:** ^1^Division of Allergy, Asthma and Rheumatology, Department of Pediatrics, Chang Gung Memorial Hospital, Taoyuan, Taiwan; ^2^Primary Immunodeficiency Care and Research (PICAR) Institute, Chang Gung University College of Medicine, Chang Gung Memorial Hospital, Taoyuan, Taiwan; ^3^Whole-Genome Research Core Laboratory of Human Diseases, Chang Gung Memorial Hospital, Taoyuan, Taiwan; ^4^Department of Microbiology and Immunology, Graduate Institute of Biomedical Sciences, College of Medicine, Chang Gung University, Taoyuan, Taiwan; ^5^Division of Hematology/Oncology, Department of Pediatrics, Chang Gung Memorial Hospital, Taoyuan, Taiwan; ^6^Division of Nephronology, Department of Medicine, Penghu Hospital, Ministry of Health and Welfare, Penghu, Taiwan

**Keywords:** CD3G, T-cell receptor, combined T and B immunodeficiency, common variable immunodeficiency, autoimmune thyroiditis

## Abstract

The T-cell receptor (TCR)/CD3 complex is crucial for T-cell development and regulation. In humans, *CD3D, CD3E*, and *CD3Z* gene defects cause severe combined T- and B-cell immunodeficiency. However, *CD3G* mutations alone lead to a less severe condition, which is mainly characterized by autoimmunity. In the present study, we report the case of a 36-year-old male who presented with recurrent sinopulmonary infections without opportunistic infections; this was compatible with hypogammaglobulinemia, but normal PHA-lymphocyte proliferation. This patient had the common variable immunodeficiency (CVID) phenotype and received regular immunoglobulin infusions over 20-years; he gradually developed nodular regenerative hyperplasia over a 5-year period. Distinct from the previously reported *CD3G* mutations, which mainly present as autoimmunity, the novel *CD3G* deletion (c.del213A) in our patient caused an obvious decrease in switched memory B cells and diminished CD40L expression. However, sufficient Treg suppression function was maintained so that he remained free of autoimmune thyroiditis (AIT), inflammatory bowel disease (IBD), and autoimmune pancytopenia. A PubMed search for this rare disease entity revealed seven Turkish and two Spanish patients (five unrelated families). Among a total of 20 alleles, there were 14 splicing mutations (80(-1)G>C), two missense mutations (c.1G>A), two nonsense mutations (c.250A>T), and two deletions (c.del213A). Three patients presented with isolated AIT without significant infections. Three patients died, one from a severe infection at 31 months, one from post-transplant respiratory failure due to viral pneumonia at 17 months, and one from graft-vs.-host disease at 47 months. Those experiencing opportunistic infections, severe life-threatening infections in need of hematopoietic stem cell transplantation, and IBD-like diarrhea had a significantly higher mortality rate compared with those without these features (*p* = 0.0124, *p* = 0.01, and *p* = 0.0124, respectively). The patients with AIT had a significantly better prognosis (*p* = 0.0124) to those without AIT. Our patient with the novel *CD3G* mutation presented with predominant B-cell deficiency overlapping with the CVID phenotype but without recognizable autoimmunity, which was consistent with his normal Treg suppression function.

## Introduction

The integrity of the T-cell receptor/CD3 (TCR/CD3) complex orchestrates T-cell maturation and activation. Before the TCR/CD3 complex reaches the membrane, TCRα/β heterodimers associate with three invariant dimers (CD3δ/ε, CD3γ/ε, and CD3ζ/ζ), which comprise the CD3 complex (1). After its localization to the cell surface, the CD3 complex mediates intracellular signaling upon antigen recognition by the TCR (2).

The complete absence of CD3δ or CD3ε chain expression has been shown to block the development of TCRα/β T cells in both humans and mice (1), and human CD3ζ deficiency has been shown to reduce the number of circulating T cells, bearing a non-functional and restricted T-cell repertoire, and thereby causing severe combined T and B immune deficiency (SCID) (3–6). CD3γ-deficient mice have a severe block in T-cell development (7), whereas the loss of the CD3γ protein in humans has been shown to allow the development of polyclonal T cells, maintain TCR/CD3 signaling, and be associated with a less severe phenotype, characterized by a varying degree of susceptibility to infection and the frequent occurrence of autoimmune disorders (8–10). Autoimmune disorders are associated with an insufficient number of Treg cells and a reduced suppression function, as well as restricted TCR diversity (11).

The present study reports a 36-year-old male adult without autoimmune manifestations who was diagnosed with common variable immunodeficiency (CVID) at 15-years old and received regular immunoglobulin infusions over the next 20-years. Whole exome sequencing (WES) was performed and revealed a novel *CD3G* deletion mutation. The CD3γ protein it coded for was truncated and lacked the ITAM domain, but global Treg cell function was unexpectedly maintained. To the best of our knowledge, this was the first patient of Chinese ethnicity to be identified with this novel *CD3G* mutation. In order to optimize his clinical management, a comprehensive review of previous phenotypes, genotypes, treatments, and prognoses was conducted by searching PubMed (8–13). The current study presents the results of this search and also discusses the effect of this novel *CD3G* deletion mutation on cellular and humoral immunity.

## Methods

### Ethics

Prior to their inclusion, the patient and healthy control provided written informed consent for the collection and publication of their data within the present study. All human samples were obtained using protocols approved by the Institutional Review Board at Chang Gung Memorial Hospital (protocols 201601893A3 and 104-9578A3) and met the Institutional Review Board standards for the ethical conduct of research with human subjects in accordance with the Declaration of Helsinki.

### Flow Cytometry for CD3, Treg and Memory T Cell Assessment, and Cell Proliferation

Peripheral blood mononuclear cells (PBMCs) were processed using Ficoll (GE Healthcare, Marlborough, MA, USA) to form a single cell suspension, which was then stained with the following monoclonal antibodies against cell surface and intracellular antigens of memory T, Treg, T follicular helper, memory B, and CD21-low B cells: anti-CD4-PE (clone SK3), CD4 FITC (clone SK3), CD8-PE (clone SK1), CD19-PerCP-Cy5.5 (clone HIB19), CD127-PE (clone hIL-7R-M21), CD25-FITC (clone 2A3), CD21-FITC (clone B-ly4**)**, IgD-PE (clone IA6-2), CD27-APC (clone M-/t271), CD45RO-PE (clone UCHL1), CD154-PE (clone TRAP1), CCR7-APC (CD197, clone 3D12), CXCR5 PerCP-Cy5.5 (clone RF8B2, all from BD Pharmingen, San Diego, CA), and FOXP3-APC (clone 236A/E7, eBioscience, San Diego, CA). Antibodies against surface CD3 (monoclonal clone SK7 PerCP-conjugated for ε chain and polyclonal OAAB01258 for γ chain) and TCRαβ-FITC (clone WT131) were used to evaluate the expression of the CD3-TCR complex in lymphocytes. For 0.5 mg/mL polyclonal antibodies to the CD3 γ chain (1:25; OAAB01258; AVIVA Bio.), the test conditions were 1 μg polyclonal antibodies in 50 μl reaction volume. Simultaneously, 3 mg/mL rabbit IgG (1:150; AB_2532981, Thermo Fisher Scientific, Inc.) was used as the isotype polyclonal control, maintaining a final concentration of 1 μg IgG in 50 μl reaction volume.

In addition to evaluating conventional lymphocyte proliferation using ^3^[H]-thymidine, as described previously (14), the expression of CD25 activation markers was evaluated in 5 × 10^5^ PBMCs. These were cultured with either medium alone, 5 mg/mL phytohemagglutinin (PHA; Sigma Aldrich, St. Louis, MO, USA), or 100 ng/mL anti-CD3 (clone HIT3a, Biolegend, San Diego, CA, USA), either alone or in combination with 100 ng/mL anti-CD28 (clone CD28.2, eBioscience) for 4 days, as described previously (15).

### Treg Suppression Assay

Treg suppressive potency was evaluated by enriching CD4+ cells through fluorescence-activated cell sorting into the following subsets: CD4+ CD25hi CD127low cells (Treg cells) and CD4+CD25low CD127hi T effector (Teff) cells. In each experiment, Teff cells were stained with 5 mM/mL carboxyfluorescein succinimidyl ester (CFSE) (Thermo Fisher Scientific, Inc., Carlsbad, CA, USA) for 10 min at 37°C in phosphate-buffered saline. Treg and Teff cells were then mixed at the indicated ratios based on a conventional CD4+ (Tconv) count of 5,000 cells per well, then stimulated with anti-CD2/CD3/CD28 beads (Treg Suppression Inspector, Miltenyi Biotec) at a 1:1 Tconv:bead ratio in complete RPMI media for 4 days. In this assay, T-cell proliferation was indicated by the dilution of CFSE.

To further analyze Treg cell suppression, PBMCs were fixed and permeabilized using a buffer (eBioscience, San Diego, CA, USA) and then stained with monoclonal antibodies against CTLA-4 (CD152, clone BNI3, BD Pharmingen, San Diego, CA, USA) after CD4 and FOXP3 staining.

### Genetic Analysis

The candidate genes for CID prone to the T-B-NK+ phenotype, including *RAG1, RAG2, DCLRE1C, PRKDC*, and *LIG4*, were all wild-type (16, 17). WES was performed using an Illumina HiSeq 2000 system (Illumina Inc., San Diego, CA, USA) on genomic DNA that was enriched for exonic fragments using an Agilent SureSelect XT Human All Exon V6 kit (Agilent Technologies, Santa Clara, CA, USA), as previously described (18, 19). The two pairs of primer sequences for the *CD3G* gene were based on human genome sequences (NM_000073.2) and were designed to cover the whole coding region with one pair: (CD3G/F-3: AGT CTA GCT GCT GCA CAG G; CD3G/R-719: CAC TTC TTG GCC GCA CCT TC). The genomic DNA in exon 3 (forward: CCA GAA CTA CTA AAT AGC ACC TG; backward: AAT TAA GAG AAC AGG CGA TAA TA) was amplified at the same time and was confirmed based on NT_033899.8 by Sanger sequencing, as previously described (20).

### Statistical Analysis

The phenotypes, genotypes, treatment, and prognosis of our patient and those identified in the PubMed search were reviewed and analyzed. The first follow-up day was defined as the age at onset, and the last follow-up day and disease duration were those reported in each study. Kaplan-Meier survival analyses were performed using GraphPad Prism software, and a *p* < 0.05 was considered to indicate a statistically significant difference.

## Results

### Case Report, Clinical Immunological, and Molecular Features

The 36-year-old male was diagnosed with CVID with hypogammaglobulinemia (Table 1), recurrent sinopulmonary infections, and a poor response to polysaccharides at 15-years of age. He was referred to our Primary Immunodeficiency Care and Research (PICAR) Institute and received regular immunoglobulin infusions over the next 20-years. He experienced recurrent sinopulmonary infections that were complicated by mastoiditis, pleural effusion, and bronchiectasis (Supplemental Figure 1A) and required hospitalization from the age of 14-years. Atrophic endobronchial mucosa caused exercise tachypnea, clubbing of the fingers, and obstructive sleep apnea. Sputum cultures commonly grew *Hemophilia influenza* or *Pseudomonas aeruginosa* despite prophylactic treatment with amoxicillin-clavulanic acid (augmentin), azithromycin, and cefuroxime. Combined isoniazid, rifampin, and pyrazinamide were administered for 1-year for the treatment of a suspected pulmonary mycobacterial infection. The patient was not admitted for pneumonia despite exacerbations during the winter. Preseptal *staphylococcus aureus* cellulitis and *E. coli* epididymo-orchitis occurred when he was 27-years-old, and an engorged portal vein, splenomegaly, splenic artery, and coarse liver surface gradually developed (Supplemental Figures 1B–D). Levels of AST, ALT, albumin, γGT, bilirubin, and αFP were measured to assess liver function, and all were within normal ranges except for a mild elevation of ALP (125 U/L, normal: 28–94 U/L). RT-PCR amplification of hepatitis virus A-E, CMV, and Epstein–Barr virus (EBV) were all negative. These findings were consistent with nodular regenerative hyperplasia (NRH). The patient favored conservative therapy and was hesitant to undergo a liver transplantation due to concerns over complications, and he was unwilling to undergo a liver biopsy for pathological proof. He was given steroid treatment (1 mg/kg/day) for 2 months in an attempt to alleviate portal vein hypertension, which may have been caused by autoimmune hepatitis, but the treatment failed. Exacerbation of his bronchiectasis increased his pulmonary artery pressure, reopened a closed atrial septal defect, and gradually progressed to cor pulmonale. He did not experience any opportunistic infections or common autoimmune disorders, such as thyroid dysfunction, hemolytic anemia, or IBD-like diarrhea. Auto-antibodies against the pancreas (insulin and islet cell antigens), thyroid (thyroglobulin, thyroid peroxidase, and TSH receptor) and liver (liver/kidney microsome [LKM], smooth muscle [SM], and mitochondria), as well as anti-phospholipid antibody syndrome were all negative, as was a Coombs test (direct and indirect).

**Table 1:** Hematological, biochemical, and immunologic evaluations of the patient.

**Age (normal reference range)**	**15 years^*$*^**	**22 years**	**27 years**	**35 years**
WBC (3,750–14,600/mm^3^)	10,500	6,700	8,920	6,800
Segment (4,500–8,500/mm^3^)	8,190	4,871	6,560	5,062
Lymphocyte (1,500–7,300/mm^3^)	1,575	1,414	1,876	1,697
Hb (>10 mg/dL)	10.8	13.8	10.2	11.7
Platelet (150/mm^3^)	348	121	152	93
AST (13–40 U/L)	10	23	24	22
ALT (<36 U/L)	9	6	10	15
Albumin (>3.5 mg/dL)	4.2		3.9	4.0
Bil D/T (<0.4/<1.3 mg/dL)	0.2/0.9	0.2/1.0	0.3/0.9	0.3/0.8
ALK-P (28–94 U/L)	32	45	97	125
γGT (10–71 U/L)	28	34	41	44
BUN (5–20 mg/dL)	9.0	8.6	8.1	7.8
Cr (0.2–1.0 mg/dL)	0.9	0.7	0.7	0.1
CRP (<5 mg/dL)	289.6	21.4	12.1	3.2
**Lymphocyte subset percentage/absolute counts (mm**^**3**^**)**				
CD3 (53.7–82.8%; 270–2,856)	78.4/1,189	58.9/833	53.2/998	58.2/988
CD4 (27.7–46.8%; 199–1,414)	47.3/745	35.0/495	32.9/617	30.8/523
CD4CD45RA (12–45%; 45–1,079)		10.7^*^/151	7.3^*^/137	3.1^*^/16^*^
CD4 T memory^∧^ (3–31%; 94–975)		22.3/315	23.7/445	19.4/101
Central (CD45ROCCR7, 9.3–27.1% of CD4; 70–671)			35.8/221	
Effector (CD45ROCCR7– 27.3–58.2% of CD4; 23–301)			45.9/283	
TEMRA (1.1–5.9% of CD4; 6–31)			1.2/7	
CD8 (8.9–29.0%; 61–1,118)	22.0/347	21.7/307	20.0/375	27.1/460
CD8 T memory^∧^ (2.4–21.6%; 20–663)			11.6/218	20.0/339
CD19 (3.8–21.5%; 51–728)		8.6/123	5.8/109	5.2/88
Memory CD19 B^∧^ (2.1–5.0%; 3–80)		3.5/4	1.7^*^/2^*^	0.02^*^/1^*^
Non-switch (CD27IgD 1.9–23.7% of CD19; 1–28)			5.9/6	
Switch (CD27IgD– 4.6–35.5% of CD19; 6–53)			0.3^*^/3^*^	
CD21low B (1.9–19.3% of CD19; 1–37)			15.5/17	17.1/15
Treg (CD4 FOXP3, 5.1–12.7% of CD4; 28–142)			5.2/32	5.4/29
Activated T cells (CD2HLADR, 4–26%; 79–1,341)		19.5/162	20.3/203	25.4/251
Natural Killer CD16CD56 cells (3–32.9%; 53–2,182)	14.2/224	29.9/423	24.1/452	22.4/380
**Immunoglobulin level**				
IgM (49–156 mg/dL)	<18.4^*^	<18.4^*^	<18.4^*^	<18.4^*^
IgG (334–1,230 mg/dL)	5.9^*^	812^#^	973^#^	1079^#^
IgG2 (30–140 mg/dL)	<2,3^*^	315^#^	342^#^	415^#^
IgA (15–113 mg/dL)	<23.1^*^	<24.6^*^	<24.6^*^	<24.6^*^
IgE (<100 IU/ml)	<17.8^*^	<17.8^*^	<17.8^*^	<17.8^*^
**Lymphocyte proliferation (cpm)**^**∧∧**^				
PHA 2.5 ug/ml (29,228–58,457)		34,765		43,257
PWM 0.1 ug/ml (11,395–42,487)		14,732		12,354
ConA 0.1 ug/ml (10,874–30,256)		5642^*^		4956^*^
Candida 2.5 ug/ml (5,351–13,328)		6,586		7,091
BCG 0.002 ug/ml (1,740–4,352)		3,091		2,967
***Pneumococcus polysaccharide response***	Negative^*^			

*Abbreviations: cpm, counts per minute; TEMRA, terminally differentiated effector memory cells re-expressing CD45RA*.

∧ *The percentage of memory CD4+ cells was calculated from multiple CD4+subsets [CD4+CD45RO+/CD4+CD45RA+ and CD4+CD45RO+] and memory CD8+ cells were calculated from multiple CD8+ subsets [CD8+CD45RO+/CD8+CD45RA+ and CD8+CD45RO+], while the percentage of memory CD19+ cells was derived from multiple CD19+ subsets [CD19+CD27+/CD19+CD27+ and CD19+CD27–]*.

* *Bold numbers represented the values below the normal ranges*.

# *The values of immunoglobulin were obtained after regular immunoglobulin infusion*.

∧∧ *For lymphocyte proliferation, PHA served as a marker of T-cell activation, ConA as a marker of B-cell activation, and PWM for both T- and B-cell activation. B-cell activation induced by ConA was lower, and lymphocyte proliferation by PWM remained borderline because normal T-cell activation induced by PWM could compensate for the defective B-cell activation induced by PWM in overall lymphocyte activation*.

$ *Normal ranges of the lymphocyte subset counts and percentages of CD4, CD4, CD8, and NK cells were based on data from our institute, Tokgoz et al. (9), Rowe et al. (11), and Kverneland et al. (27)*.

**Figure 1:**
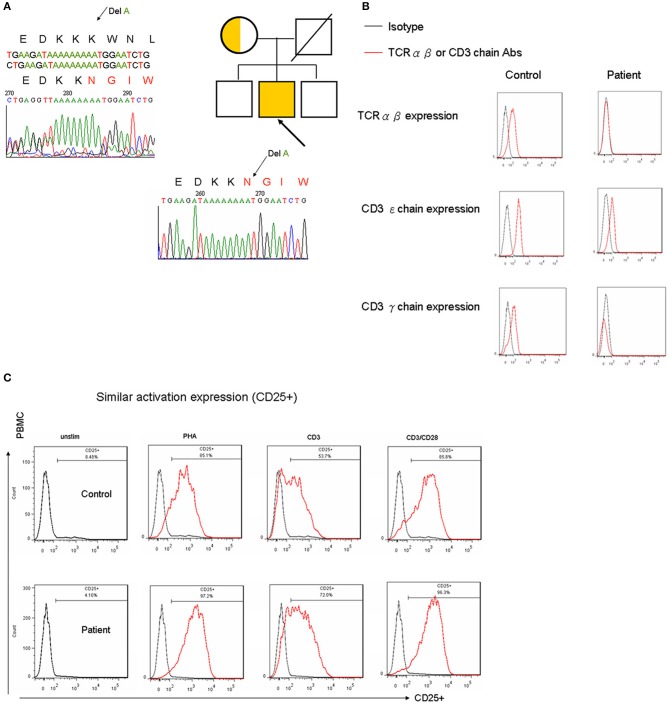
**(A)** Whole-exome Sanger sequencing mutually identified an A deletion that caused N to be substituted by K at the 71st amino acid; this interrupted the last two amino acids of the immunoreceptor signaling ITAM domain and truncated at the following 39 location in the *CD3G* gene. **(B)** Following CD4+ gating for surface CD3-TCR complex expression, CD3ε chain expression decreased by one-log fluorescence intensity, and the γ chain of the CD3 complex was almost undetectable. There was an obvious decrease in TCRαβ in the patient with a CD3γ^Del^ ^213A^ mutation. Similar results were obtained following CD8 gating (data not shown). The units of the y-axis are cell counts between 0 and 200. **(C)** 5 × 10^5^ PBMCs per well were cultured with 5 mg/mL phytohemagglutinin (PHA) or 100 ng/mL anti-CD3, either alone or in combination with 100 ng/mL anti-CD28, for 4 days. The lymphocyte proliferation staining with the activation marker for CD25 in flow cytometry was similar between the patient and the healthy control.

The observed T-B-NK+ SCID presentation was not supported by the patient's lymphocyte proliferation (Table 1), which was consistent with wild-type candidate genes (17). Using a WES approach (Supplemental Table 1), a 1 base pair deletion was identified at position 71 in the coding sequence (c.213 del A), which led to a frameshift at position 71 and a premature stop codon at position 110 (Figure 1A).

### Decreased Surface CD3/TCRαβ Expression but Normal Lymphocyte Proliferation in the CD3γ^Del^ ^213A^ Mutation

To determine the impact of *CD3G* mutations on the expression of the TCR/CD3 complex, CD4+ and CD8+ T cells from the patient and controls were analyzed, as shown in Figure 1B. It was found that the expression of CD3 (as indicated by staining with a monoclonal antibody specific for the CD3ε chain and polyclonal Abs for the CD3γ chain) decreased by one-log fluorescence intensity. The expression of TCRαβ also clearly decreased in the patient compared with the control, which did in agreement with previous studies (8, 9, 11, 12).

In keeping with the normal PHA-lymphocyte proliferation assessed by ^3^[H]-thymidine (Table 1), cellular activation as determined by CD25 staining under stimulation with PHA, CD3, and CD3/CD28, was consistently similar between the healthy control and the patient (Figure 1C). However, defective B-cell activation, induced by ConA and incorporated with ^3^[H]-thymidine, was identified.

### Normal Treg Suppressive Activity With the CD3γ^Del^ ^213A^ Mutation

To explain and predict whether a patient with the CD3γ^Del^ ^213A^ mutation could develop an autoimmune phenotype (9, 10, 12), the development and function of Treg cells were investigated in the patient. Among his CD4+ T cells, the proportion of intracellular FOXP3+ Treg cells was similar to that observed in the control (Table 1).

**Figure 2:**
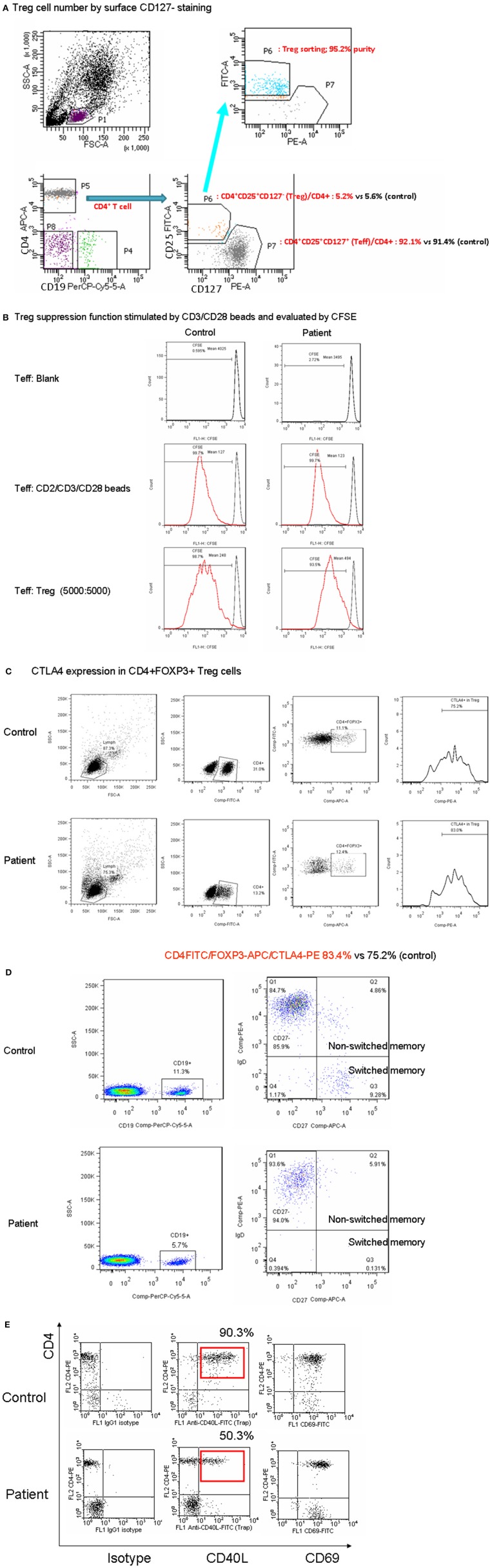
**(A)** The patient with CD3γ^Del^ ^213A^ deficiency had a normal number of Treg cells in intracellular FOXP3 (Table 1) and similar CD4+CD25+CD127-staining compared with the control in CD4+ cells. **(B)** When co-cultured with T effector and Treg cells using 5000:5000 and stimulation by CD2/CD3/CD28 beads to assess Treg suppression, the percentage of CFSE shifted to the left (99.7 to 93.5% vs. 99.7 to 98.7% in the control) and the mean fluorescence intensity (494 to 123% vs. 248 to 127% in the control) showed a similar pattern. **(C)** Furthermore, the critical CTLA4 expression of Treg cells for suppression was within the normal range (83.4 vs. 75.4% in the control). **(D)** In the patient with CD3γ^Del^ ^213A^ deficiency, memory B cells (CD19+CD27+) decreased (6.8 vs. 13.2–48.7% in the control), especially class-switched memory cells (CD19+CD27+IgD– 0.1 vs. 9.2–39.2% in the control). **(E)** The CD40L expression of activated CD4+ in the patient was around half that of the healthy control, while CD69 expression was used as an activation marker after stimulation with 10 ng/mL PMA and 1 ug/mL ionomycin for 5 h. Two duplicates were performed.

It was then investigated whether the decrease in CD3 expression observed in CD3γ^Del^ ^213A^ T cells would inhibit Treg cell suppression of Tconv cell activation. CD4+ T cells from the patient and the control were sort-purified into CD25hi CD127low Treg and CD25low CD127hi Teff cells (Figure 2A). The Teff cells were then assayed for cell division following coculture with a 1:1 ratio of Treg cells isolated from either the control or the patient. The percentage of dividing Teff cells upon stimulation with anti-CD2/CD3/CD28 beads in the absence and presence of Treg cells revealed a similar suppression of cell proliferation to the Treg cells at a final Treg:Teff cell ratio of 1:1 (Figure 2B). In addition, CTLA4 expression, which leads to the suppression of Treg cells, reached a normal level in the patient (Figure 2C). Taken together, these findings demonstrate that Treg cells isolated from the CD3γ^Del^ ^213A^ T cells of the patient were able to sufficiently suppress the proliferation of Teff cells upon stimulation, indicating that this normal Treg suppressive function may prevent the development of autoimmunity.

### Lower Switch Memory B Cells but Normal CD21-Low B Cells in the CD3γ^Del^ ^213A^ Mutation

The patient had normal memory T (CD4+CD45RO+), T follicular helper (Tfh; CD4+CXCR5+), and lowCD21 B cells (Supplemental Figure 2) but a mild decrease in naïve T cells (CD4+CD45RA+ or CD4+CD45RO-CCR7+; Table 1 and Supplemental Figure 2). His CD27+ memory B cells were obviously decreased (6.8%), predominantly in switched CD27+ memory B cells (CD19+IgD+CD27+; 0.1%; Figure 2D). Because intact CD40-CD40L signaling enhanced the maturation of memory B cells, a diminished expression of CD40L in activated CD4+ cells in the patient (Figure 2E) inhibited the maturation process, especially in class-switch memory B cells.

### Genotype, Phenotype, and Survival Analysis

**Figure 3:**
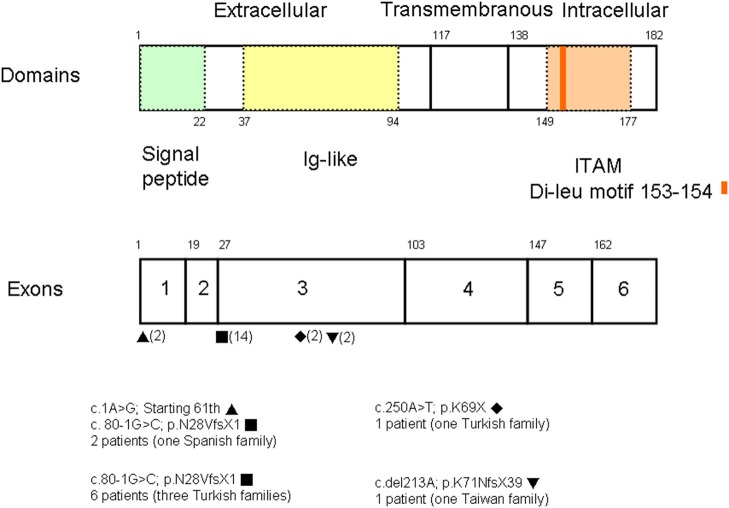
Among a total of 20 alleles, there were the splicing mutation c.80-1G>C in 14, the missense mutation c.1G>A in two, the nonsense mutation c.250A>T in two, and the deletion c.del213A in two. Based on the uniprot/P09693 structure, our patient with the homozygous A deletion located in the Ig-like domain had lost critical transmembranous (117–137 amino acids) and intracellular components (138–182 amino acids).

A PubMed search using the keywords “*CD3G* mutation” and “immunodeficiency” revealed reports of seven patients of Turkish descent and two of Spanish descent from five unrelated families (three with consanguinity) (8–12). Studies that did not identify the gene of interest were excluded. Among a total of 20 alleles (Figure 3), the splicing mutation c.80-1G>C was found in 14, the missense mutation c.1A>G in two, the nonsense mutation c.250A>T in two, and the deletion c.del213A in two. The founder effect of c.80-1G>C seemed to exist in patients of both Turkish and Spanish descent.

Autoimmune disorders were the most common manifestation, seen in nine patients, followed by infections, in seven patients (Table 2). The autoimmune-related disorders included autoimmune thyroiditis (six patients), IBD-like diarrhea (four patients), and autoimmune hemolytic anemia (four patients). Four of the five patients with recurrent sinopulmonary infections developed bronchiectasis. Three patients without significant infections presented with isolated autoimmune thyroiditis that was associated with antibodies against thyroglobulin, thyroid-stimulator hormone receptor, and/or peroxidase. Only our patient with CD3γ^Del^ ^213A^ had neither autoimmune disorders nor any detectable autoantibodies. Patients with opportunistic infections, including parainfluenza pneumonia, *Candida albicans, Giardia intestinalis*, and severe EBV infection, reflect profoundly impaired lymphocyte proliferation and become candidates for transplantation (8, 11). There were three fatalities, one from a severe infection at 31 months (8), one from post-transplant respiratory failure at 17 months, suspected of being caused by viral pneumonia (11), and one from post-transplant graft-vs.-host disease at 47 months (13).

**Table 2:** Clinical features and immunological defects in 10 patients with CD3G mutations.

**Characteristic**	**Total**
**Onset age** **<1 year**	**7**
**>1 year**	**3**
**Patient ethnicity** (families)	**10** (6)
Turkish	**7** (4)
Spanish	**2** (1)
Taiwanese	**1** (1)
**Consanguineous** (Turkish)	**1**
**Autoimmune-related**	**9**
Autoimmune thyroiditis	6
Inflammatory bowel disease (IBD) or IBD-like diarrhea	4
Autoimmune hemolytic anemia	4
Vitiligo	2
Minimal change nephrotic syndrome	1
Granulomatous lymphocytic interstitial lung disease	1
Dilated cardiomyopathy	1
Autoimmune hepatitis	1
**Infection**	**7**
Recurrent sinopulmonary infection	5
Opportunistic infections^*^	4
Soft tissue abscess	2
Severe varicella	1
Giardia intestinalis	1
Viral meningitis	1
**Failure to thrive**	**4**
**Bronchiectasis**	**4**
**Hepatosplenomegaly**	**2**
**Osteoporosis**	**2**
**Significant immunologic anomalies**	
Decreased lymphocyte proliferation	**8**
Mild (without opportunistic infections)	5
Profound (with opportunistic infections)	3
Low naïve CD4+CD45RA+ percentage	**7**
Selective deficiency to polysaccharide	**5**
Low IgG and/or IgG2 immunoglobulin	**5**
**Treatment**	
Immunosuppressants	**6**
Prophylactics (for bronchiectasis)	**5**
Regular IVIG	**4**
HSCT	**2**
**Mortality**	**3**
Respiratory failure to severe pneumonia	1
Post-transplant GvHD	1
Post-transplant respiratory failure	1

* *Opportunistic infections include parainfluenza pneumonia, candidiasis, severe EBV infection in each and Giardia intestinalis and candidiasis in one*.

In terms of cellular phenotypes, a one-log lower fluorescent density of CD3 expression and almost undetectable TCRαβ expression were noted in all patients. A lower percentage of CD4+CD45RA+, mildly impaired lymphocyte proliferation, hypogammaglobulinemia, lower IgG2, and selective deficiency to polysaccharides were noted in some patients but not all (Supplemental Table 2).

**Figure 4:**
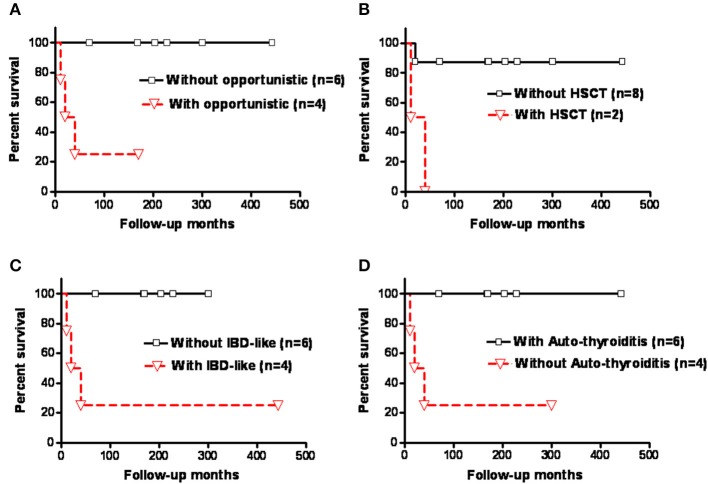
Kaplan-Meier survival analysis of the patients with *CD3G* mutations showed that those who had **(A)** opportunistic infections, **(B)** severe life-threatening infections requiring HSCT, and **(C)** IBD-like diarrhea had a significantly higher mortality rate than those without (*p* = 0.0124, *p* = 0.01, and *p* = 0.0124, respectively). **(D)** Patients with autoimmune thyroiditis had a significantly better prognosis (*p* = 0.0124). Y-axis indicated patient survival rate (100%).

Kaplan-Meier survival analysis showed that those who had opportunistic infections, severe life-threatening infections in need of hematopoietic stem cell transplantation (HSCT), and IBD-like diarrhea had significantly higher mortality rates compared with those without (*p* = 0.0124, *p* = 0.01, and *p* = 0.0124, respectively; Figures 4A–C). The patients with autoimmune thyroiditis had a significantly better prognosis compared to those without (*p* = 0.0124; Figure 4D), but this was not observed for autoimmune hemolytic anemia (AIHA) (*p* = 0.3581; Supplemental Figure 3 and Supplemental Table 3).

## Discussion

The identified novel homozygous A deletion in the *CD3G* gene coded for proteins lacking trans-membrane and intracellular ITAM components and mainly led to B-cell deficiency, especially in class-switch CD27+ memory B cells. This may have been associated with insufficient CD40L expression, which has been reported in some patients with CVID and hyper IgM (20–23). The number of Tfh cells capable of a robust vaccine response was within normal range. Despite a mild reduction in naïve T cells, the patient's T-lymphocyte proliferation ability was sufficient to prevent opportunistic infections. The normal Treg count and CTLA-4 expression maintain adequate Treg suppression to prevent autoimmune disorders.

Mutations in CD3δ (CD3D), CD3ε (CD3E), and CD3ζ (CD247) cause the T-B+NK+ SCID phenotype, resulting in profound lymphocyte impairment and increased susceptibility to opportunistic infections, in need of rescue by HSCT. The symptoms are less severe in patients with *CD3G* mutations, and only two (20%) of all 10 published cases received HSCT due to refractory IBD, AIHA, and granulomatous lymphocytic interstitial lung disease or severe life-threatening infections. One of these two transplant patients, who had a p.K69X mutation, died of post-transplant respiratory failure (14), and the other, with a p.N28VfsX1 mutation, died of severe graft-vs.-host disease (11). Four of the other seven surviving patients received regular immunoglobulin infusions for hypogammaglobulinemia and bronchiectasis. Four of the five patients with autoimmune thyroiditis received thyroxine. The immunosuppressant therapy used for autoimmune disorders mainly included steroids, cyclosporine, mesalazine, sirolimus, and rituximab in patients with IBD-like diarrhea, AIHA, nephritic syndrome, autoimmune hepatitis, and granulomatous lymphocytic interstitial lung disease. Taken together, the patients with *CD3G* mutations were characterized by predominant B-cell deficiency and autoimmune hypothyroiditis, except for our CD3γ^Del^ ^213A^ patient, who did not develop autoimmune disorders.

NRH is not uncommon in patients with predominant B-cell deficiency, including CVID, X-linked agammaglobulinemia, and hyper IgM patients (24, 25). Based on the clinical course of NRH in patients with CVID, three classifications have been proposed: (I) non-progressive and not causing clinical liver disease, (II) slowly progressing to portal hypertension and splenomegaly causing mild ALP elevation, and (III) more rapidly progressing and combined with autoimmune hepatitis-like syndrome (25). Vascular engorgement of the portal vein, uneven liver surface, tortuous splenic artery, splenomegaly, and poor response to steroids were characteristic of NRH classification II in our patient with the CD3γ^Del^ ^213A^ mutation (26). This presentation of NRH expands the phenotypic diversity of the few patients with *CD3G* mutations who have an obvious decrease in class-switch memory B cells and meet the criteria for the CVID phenotype, as in the Paris MB0 classification (6.8% CD27 of total B cells <11%), Freiburg Ib classification (0.1% switched CD27IgD– of total B cells <0.4% and 15.5% CD21 low of total B cells <20%), and EuroClass group smB-CD21low (0.3% switched memory cells ≤2% and 15.5% CD21lowB cells >10%) (27).

There were some limitations to the current study. First, only 10 patients with *CD3G* mutations have been identified to date. Hypogammaglobulinemia, IgG2 subclass deficiency, recurrent sinopulmonary infections, and autoimmune disorders overlap with the CVID phenotypes. Therefore, *CD3G* could be a candidate gene for a subgroup of CVID patients. Second, *CD3G* mutations have mostly been identified in seven Turkish patients from four families. Although geographic distribution could be an explanation, the real numbers may have been underestimated, and this should be validated in a large cohort of patients with the CVID phenotype. Third, the phenotypic spectrum caused by the same mutation in the same family was diverse; it ranged from asymptomatic to severe T-cell deficiency requiring HSCT. Further investigation into the epigenetic effect and gene–gene modification relationships are needed to elucidate the compensatory mechanisms at play. Fourth, in addition to normal numbers of Tfh and Treg cells and Treg suppression, an in-depth investigation of the co-culture of the Tfh/B-cell system is helpful to evaluate autocrine (IL-21) and the development of memory B and plasma cells. High-throughput sequencing for the TCR repertoire in Treg cells would further verify the phenotype in the absence of autoimmune disorders at a genetic and molecular level.

In conclusion, our patient had a novel *CD3G* gene deletion and presented with predominant B-cell deficiency and NRH, which mimicked the CVID phenotype but without the occurrence of autoimmune disorders; this was consistent with his normal number of Treg cells and his normal suppression function. Overall, the patients identified in the PubMed search without IBD-like diarrhea or opportunistic infections and those with autoimmune thyroiditis had better survival and were all alive at the end of follow-up. Only two patients who had severe IBD and recurrent opportunistic infections received HSCT, but they succumbed to complications.

## Data Availability Statement

All datasets generated for this study are included in the article/Supplementary Material.

## Ethics Statement

The studies involving human participants were reviewed and approved by The patient and the healthy controls had provided written consent for publication of this study. All human samples were obtained under protocols approved by the Institutional Review Boards at Chang Gung Memorial Hospital (protocol 201601893A3 and 104-9578A3) and met the Institutional Review Board standards for ethical conduct in accordance with the Declaration of Helsiniki. The patients/participants provided their written informed consent to participate in this study. Written informed consent was obtained from the individual(s) for the publication of any potentially identifiable images or data included in this article.

## Author Contributions

W-IL and J-LH carried out the molecular genetic studies, participated in the sequence alignment, and drafted the manuscript. C-HL and M-LK performed Treg functions. W-IL and W-LF conducted the sequence alignment. W-IL, C-HL, and J-LH designed the study and performed statistical analysis. S-HC, S-JL, L-CC, T-CY, W-ST, and T-HJ took care of the patient's critical condition. W-IL conceived the study and coordinated the investigation. All authors read and approved the final manuscript.

### Conflict of Interest

The authors declare that the research was conducted in the absence of any commercial or financial relationships that could be construed as a potential conflict of interest.
