# Supplementary material for: A Novel CD3G Mutation in a Taiwanese Patient With Normal T Regulatory Function Presenting With the CVID Phenotype Free of Autoimmunity—Analysis of all Genotypes and Phenotypes
Source: Front Immunol. 2019 Dec 19;10:2833. doi: 10.3389/fimmu.2019.02833 (PMC6930882; doi:10.3389/fimmu.2019.02833)

Supplemental Table 1. List of deleterious variants found by the WES analysis

Gene	Chr	Position	Ref	Var	Type	Nucleotide change	A.A. Change	snp150	1000G_ALL	1000G_EAS	ExAC_ALL	ExAC_EAS	SIFT	Polyphen2	MutationTaster	FATHMM	M-CAP	CADD score
PGM1	1	64101908	C	T	nonsense	c.C931T	p.R311X	rs377295149	.	.	0	0	.	.	DCA	.	.	45
HRNR	1	152191701	A	T	missense	c.T2404A	p.S802T	rs143461895	.	.	0	0.0005	D	D	PN	T	T	0.145
IL10	1	206945660	G	A	missense	c.C121T	p.L41F	rs750010814	.	.	0	0.0001	D	D	DC	D	D	25.6
CENPF	1	214791992	C	T	nonsense	c.C436T	p.Q146X	rs753435122	.	.	0	0	.	.	DCA	.	.	39
GEN1	2	17961356	A	C	missense	c.A1376C	p.Q459P	rs763725122	.	.	0	0.0002	D	P	DC	T	D	23.8
XDH	2	31595127	A	C	missense	c.T1823G	p.L608R	rs757717669	.	.	0	0.0003	D	D	DC	T	D	28.9
MZT2B	2	130948080	G	C	missense	c.G358C	p.A120P	rs770888686	.	.	0	0.0003	T	P	PN	T	T	7.7
RND3	2	151326608	A	G	missense	c.T628C	p.S210P	rs778598169	.	.	0	0.0002	D	P	DC	T	T	21.7
TTN	2	179518001	C	T	missense	c.G38755A	p.A12919T	rs781453248	.	.	0.0004	0.0007	.	.	PN	T	T	12.26
ITGAV	2	187519408	G	A	missense	c.G1429A	p.G477S	rs202013444	.	.	0	0.0002	D	D	DC	T	T	31
FN1	2	216236888	G	A	missense	c.C5915T	p.P1972L	rs199867755	0.0002	0.001	0	0.0001	T	D	DC	T	T	22.8
LRRFIP1	2	238617232	C	T	missense	c.C142T	p.R48C	rs774712031	.	.	0	0.0002	D	D	DC	T	D	28.6
NUP210	3	13364899	G	A	missense	c.C4678T	p.R1560C	rs751776029	.	.	0	0.0006	T	D	DC	T	T	23.2
SCN11A	3	38945411	A	G	missense	c.T1787C	p.M596T	rs758216987	.	.	0	0.0002	D	P	DC	D	D	23.5
EIF4G1	3	184045222	G	A	missense	c.G3062A	p.R1021H	rs34086109	0.0036	.	0.0012	0	D	D	DC	T	.	34
PRSS12	4	119204219	T	C	missense	c.A2087G	p.H696R	rs772219645	.	.	0	0.0001	D	D	DC	D	D	23.3
MFSD8	4	128865090	C	T	missense	c.G256A	p.G86S	rs552923962	0.0002	0.001	0.0001	0.001	D	D	DC	T	D	31
HK3	5	176309033	C	T	missense	c.G2149A	p.A717T	rs761890560	.	.	0	0.0003	D	D	DC	D	D	34
GRM6	5	178413676	G	A	missense	c.C1579T	p.R527W	rs149199617	0.0002	0.001	0.0003	0.0006	D	D	DC	D	D	29.5

C6orf222	6	36294383	C	T	missense	c.G940A	p.A314T	rs565352938	.	.	0.0001	0.001	D	D	PN	T	T	13.37
ADGRF2	6	47650352	A	G	missense	c.A1853G	p.N618S	rs372180424	.	.	0	0	D	D	DC	T	T	25.1
PKHD1	6	51735334	A	G	missense	c.T7454C	p.I2485T	rs760835535	.	.	0	0.0006	D	D	DC	D	D	28.4
MUC12	7	100643689	C	G	missense	c.C9845G	p.T3282R	rs200604108	.	.	0.0005	0	D	.	PN	T	T	10.44
SSPO	7	149517983	G	A	missense	c.G12326A	p.G4109D	rs755087339	.	.	0	0.0002	.	D	.	.	.	8.076
AGAP3	7	150820911	A	G	missense	c.A475G	p.K159E	rs760342351	.	.	0	0.0001	T	P	PN	T	D	14.36
ESYT2	7	158529799	C	T	missense	c.G2420A	p.R807Q	rs556761451	0.0002	.	0	0.0003	T	D	DC	T	T	31
RBM12B	8	94745998	T	G	missense	c.A2641C	p.S881R	rs770401634	.	.	0	0.0001	T	P	PN	T	T	15.53
VLDLR	9	2645061	A	G	missense	c.A1291G	p.T431A	rs201031743	0.0004	0.002	0	0.0007	D	P	DC	D	D	25
FBP1	9	97367843	A	G	missense	c.T721C	p.Y241H	rs757338386	.	.	0.0001	0.0006	D	P	DC	T	D	24.6
SETX	9	135205295	A	C	missense	c.T1690G	p.L564V	rs761877146	.	.	0	0.0007	D	D	PN	D	D	23.7
RABL6	9	139734210	C	T	missense	c.C1826T	p.P609L	rs373963047	.	.	0	0	T	P	PN	T	D	8.937
SYT15	10	46967503	C	T	missense	c.G574A	p.D192N	rs782157420	.	.	0	0.0005	.	D	DC	.	T	28.6
KBTBD3	11	105923748	T	A	missense	c.A1668T	p.L556F	rs768728942	.	.	0	0.0001	D	D	DC	T	D	23.2
CD3G	11	118220583	A	-	Deletion	c.205delA	p.K69fs	rs570768621	0.0006	.	0.0004	0.0007	.	.	.	.	.	.
KDM5A	12	442670	T	C	missense	c.A1636G	p.M546V	rs767612195	.	.	0	0.0001	D	D	DC	T	D	24.7
RITA1	12	113629192	C	T	missense	c.C452T	p.P151L	rs753990128	.	.	0	0.0002	D	D	PN	T	D	24.5
RNF17	13	25424571	T	A	missense	c.T3194A	p.V1065E	rs573781576	0.0002	0.001	0.0001	0.0007	T	D	DC	T	T	4.456
ZC3H13	13	46544556	G	A	missense	c.C2513T	p.P838L	rs753228361	.	.	0	0	D	D	DC	T	D	24.2
OR4N2	14	20296217	G	A	missense	c.G610A	p.G204S	rs768765567	.	.	0.0001	0.0006	D	D	PN	T	T	25.7
FAM71D	14	67671475	C	T	missense	c.C581T	p.T194M	rs150115768	.	.	0	0	.	D	.	.	.	24.7
HEATR4	14	73989367	G	A	missense	c.C490T	p.L164F	rs775159885	.	.	0	0.0001	T	P	PN	T	T	0.456
AHSA1	14	77931991	G	A	missense	c.G671A	p.R224K	rs774555249	.	.	0	0.0005	T	D	DC	.	T	24.8

VPS13C	15	62210389	C	T	missense	c.G7577A	p.R2526H	rs771908102	.	.	0	0	T	P	PN	T	T	22.2
TLN2	15	63068996	G	A	missense	c.G5401A	p.A1801T	rs141816570	0.0002	.	0.0002	0.0001	T	P	DC	T	T	23.5
CD276	15	73994910	G	A	missense	c.G394A	p.A132T	rs777380321	.	.	0	0	T	D	DC	T	T	22.8
RNMTL1	17	686349	G	C	missense	c.G341C	p.R114T	rs761902936	.	.	0	0.0003	D	D	DC	T	D	25.8
CFAP52	17	9515730	C	T	missense	c.C755T	p.T252M	rs376260961	.	.	0	0.0001	T	D	DC	T	D	23.4
AATF	17	35348134	A	T	missense	c.A1376T	p.D459V	rs754309759	.	.	0	0.0007	.	D	DC	.	D	32
FAM104A	17	71205676	G	-	nonsense	c.553delC	p.L185X	rs752301913	.	.	0	0.0006	.	.	.	.	.	.
RHBDF2	17	74473818	C	T	missense	c.G722A	p.R241H	rs149960669	.	.	0	0.0001	D	D	DC	T	D	32
CDH2	18	25572690	C	T	missense	c.G1180A	p.G394R	rs201382169	.	.	0	0.0002	D	D	DC	T	D	33
ZC3H4	19	47593350	C	T	missense	c.G589A	p.E197K	rs748445260	.	.	0	0.0005	T	P	DC	T	T	29.2
ITSN1	21	35247686	C	T	missense	c.C4202T	p.P1401L	rs756814425	.	.	0	0	D	D	DC	T	T	24.3
COL18A1	21	46924434	A	T	splicing site	c.2825-2A>T		.	.	.	0.0004	0	.	.	.	.	.	.
CELSR1	22	46794497	G	A	missense	c.C5450T	p.T1817M	rs145851305	0.0008	.	0.0005	0.0001	T	D	PN	T	D	20.4

Abbreviations: 1000g: 1000 genome project; ExAC: The Exome Aggregation Consortium; EAS, East Asian; D: Deleterious; T: Tolerated; P: Possibly damaging; B: benign; N: Neutral; U: Unknown; DCA: disease causing automatic; DC: disease causing; PN: polymorphism (probably harmless); PA: polymorphism automatic (known to be harmless).

The variations were reported as a tolerated mutation in Polyphen2 or in the 1000 Genomes Project with minor allele frequency (MAF) ≥0.001 were excluded.

Supplementary Table 2. Clinical features, genotypes and immunophenotypes of published patients with CD3G mutations

Mutation point	Ethnicity	Mutation type			Onset/ Gender	Immunophenotype: Low CDD4, CD8 but normal B cell number				Significant events			Published Survival[Mortality cause] (alive age)	Published year [Ref.]
		Exon	type	domain		T cell		B cell		Infection	Autoimmune	Others (HSCT)		
						CD45 RA	Prolife-ration	Igs level	Response					
c. 1 A>G Starting 61th<br>c. 80(-1) G>C N28V Fs 1 X	Spanish (non-consanguineous)	1<br>3	Missense<br>Splicing	Ig-like<br>Signal, Transmembrane, Transmembrane helix	11M/M	↓	↓	IgG2↓	Polysaccharide ↓	Parainfluenza, H. influenza pneumonia	IBD, AIHA (Abs to mitochondria, smooth muscle, intestinal epithelia), giant cell Hecht's pneumonia	Failure to thrive	[31M]	1992 [8]
					1M/M	↓	↓ (mid)	IgG↓IgG2↓	Polysaccharide ↓	Soft tissue abscesses, viral meningitis	IBD-like, vitiligo, antithyroglobulin, anti-thyroid peroxidase [Thyroxine]	Asthma, eczema, dilated cardiomyopathy	37Y	1992 [8], 2000 [12], 2018 [11]
c.250A>T; p. K 69 X	Turkish (non-consanguineous)	3	Nonsense		6M/ M	↓	↓(mild)	N	Normal	Recurrent pneumonia, candidiasis	IBD (fistula) [Tx: steroids, cyclosporine, mesalazine, HSCT]	Failure to thrive; HSCT (7M) Family history: early death [11M]	17 M [respiratory failure]	2008 [14]
c. 80(-1) G>C N28V Fs 1 X	Turkish (non-consanguineous)	3	Splicing	Signal, Transmembrane, Transmembrane helix	1Y/M	↓	↓	IgG↓ IgA↓	Normal	Recurrent pneumonia, bronchiectasis	Evan syndrome [Tx: steroids, cyclosporine A, IVIG], AIHA (positive Coombs 3+), autoimmune hepatitis, thyroiditis, minimal change nephritic syndrome [Tx: steroids]	Hepatosplenomegaly	18Y	2013 [9], 2018 [11]
					10Y/M	↓	↓(mild)	N	Normal		Autoimmune thyroiditis [thyroxine], AIHA		24Y	
c. 80(-1) G>C N28V Fs 1 X	Turkish (consangui	3	Splicing	Signal, Transmem	10M/F	↓	↓(mild)	IgG↑ IgE↑	Normal	Chronic sinusitis, Recurrent pneumonia,	Diffuse vitiligo, autoimmune hyperthyroidism (anti-thyroglobulin, microsomal,	Failure to thrive, osteoporosis, hearing impairment; plan to	15Y	2014 [10]

	neous)			brane, Transmembrane helix						bronchiectasis, severe varicella (3Y), Giardia intestinalis, candida albicans	anti-TSHR Abs and ANA) [thyroidectomy]	HSCT		
					11M/M	↓	↓(mild)	IgE↑	Normal		autoimmune hyperthyroidism (anti-thyroglobulin, microsomal Ab), pityriasis alba, ANA	Failure to thrive, Atopic dermatitis, osteoporosis	19Y	2014 [10], 2018 [11]
					14M/F	↓	↓(mild)	N	Normal		autoimmune hypothyroidism	Dx by family screening	6Y	
c. 80(-1) G>C N28V Fs 1 X	Turkish (non-consanguineous)	3	Splicing	Signal, Transmembrane, Transmembrane helix	7M/M	Normal	↓	IgG↓ IgM↓	Polysaccharide ↓	Klebsiella pneumonia, MSSA, severe EBV	Autoimmune enteropathy (IBD-like), Granulomatous lymphocytic interstitial lung disease, AIHA	HSCT (32M)	[47M], [GvHD, infections]	2018 [11]
c. Del 213A, p. K71N Fs 39 X	Taiwan (non-consanguineous)	3	Deletion	Ig-like, Signal, Transmembrane, Transmembrane helix	11Y/M	↓	**N**	IgG↓ IgM↓ IgA↓	Polysaccharide ↓	Recurrent pneumonia, chronic sinusitis, bronchiectasis	No	Splenomegaly, nodular regenerative hyperplasia	36Y	This study

↓ indicates lower than normal ↑ indicates higher than normal; Gray background indicates mortality.

Abbreviations: IBD, inflammatory bowel disease; AIHA: autoimmune hemolytic anemia; IVIG, intravenous immunoglobulin; HSCT, hematopoietic stem cell transplantation; Tx: treatment

Supplemental Table 3: Hazard ratios of the patients with *CD3G* mutations

Patient status*	Hazard Ratio	95% CI of Ratio	Significance
Receiving HSCT	0.08505	0.0005651 to 0.3614	Yes
Opportunistic infection	0.0000	0.003944 to 0.5105	Yes
IBD-like diarrhea	0.0000	0.003944 to 0.5105	Yes
AIHA	0.3424	0.03371 to 3.406	No

*Autoimmune thyroiditis was included.

Abbreviations: HSCT, hematopoietic stem cell transplantation; IBD, inflammatory bowel disease; AIHA, autoimmune hemolytic anemia

Supplemental Figure legends:

Figure 1. Recurrent sinopulmonary infections led to bronchiectasis (A). Coarse liver surface and heterogeneous cirrhosis-like parenchyma were found on an ultrasound (B). Hepatomegaly with an uneven surface, engorged portal vein, splenomegaly and tortuous splenic artery were also observed (C and D).

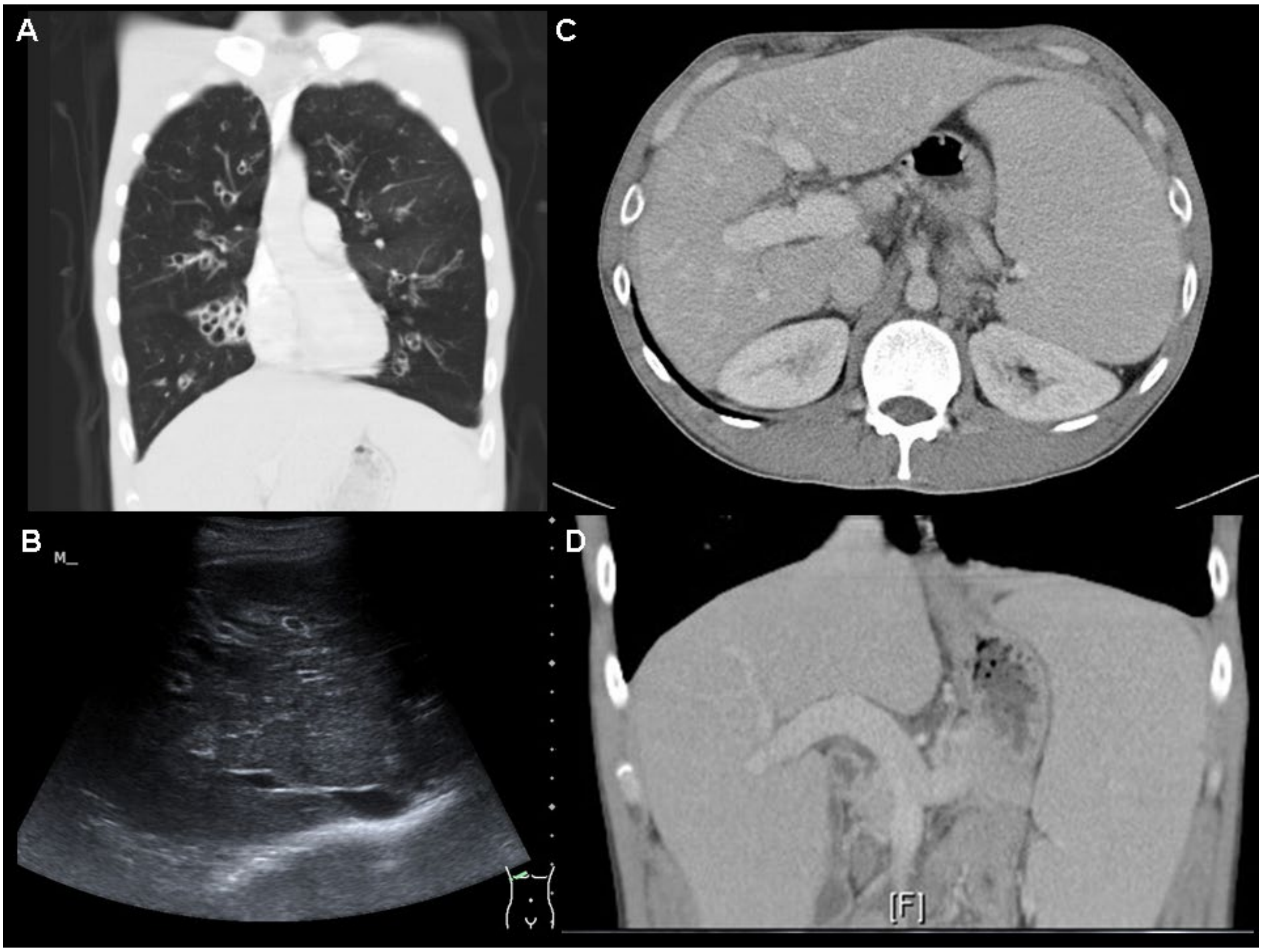

Figure 2. Under CD4 gating, the patient had normal T follicular help cells (Tfh: CD4+CXCR5+) 16.7%, which was within the normal range (8.0-23.7%) (A). Except for a mild decrease in naïve T cells (17.2%, normal range: 25.2-53.8%), the other populations of central memory, effector memory and T_{EMRA} cells were all within normal ranges (35.8%, 45.9% and 1.2% in CD4+ T cells) (B). Normal CD21low B cell was 15.5% within the normal range (as in Table 1) (C).

Suppl. Fig 2

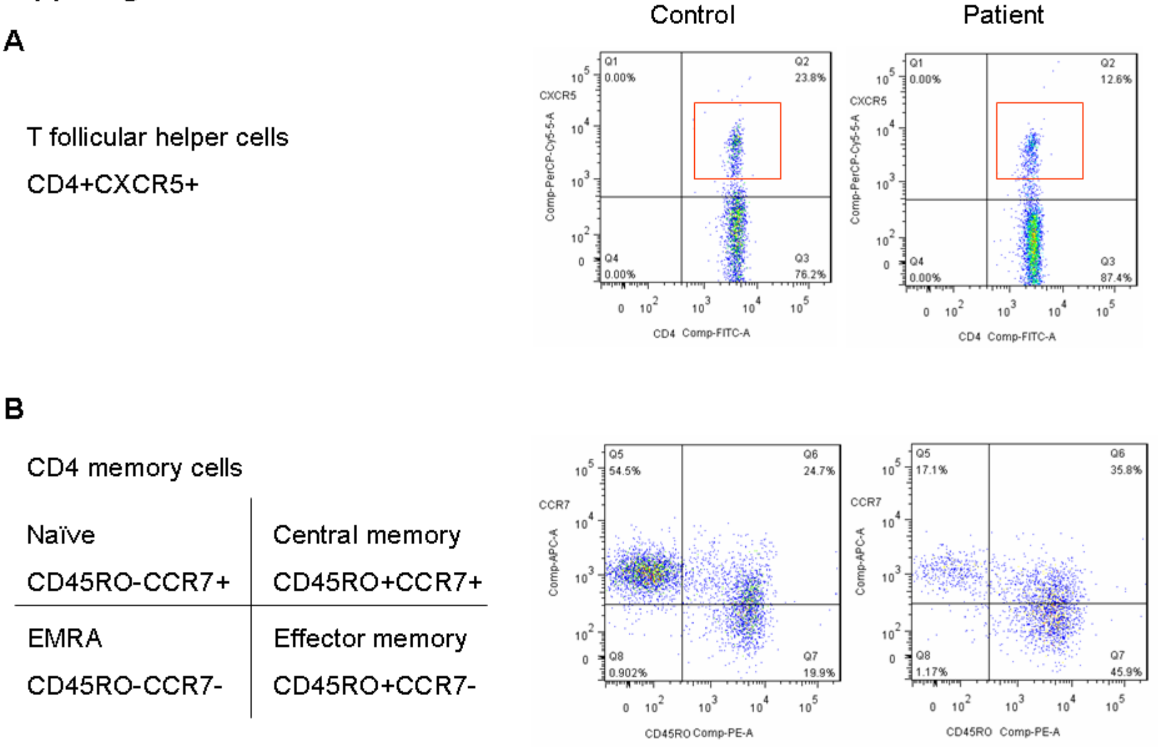

EMRA: terminally differentiated effector memory re-expressing CD45RA

Suppl. Fig 2

C

CD21low B cell

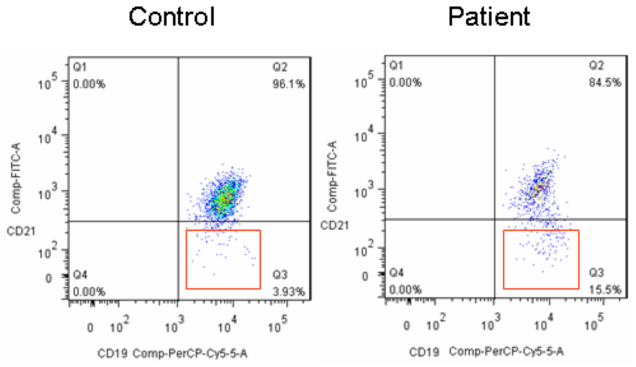

Figure 3. Patients without autoimmune hemolytic anemia (AIHA) tended to have better survival than those AIHA, although the difference was not significant (p=0.3581).

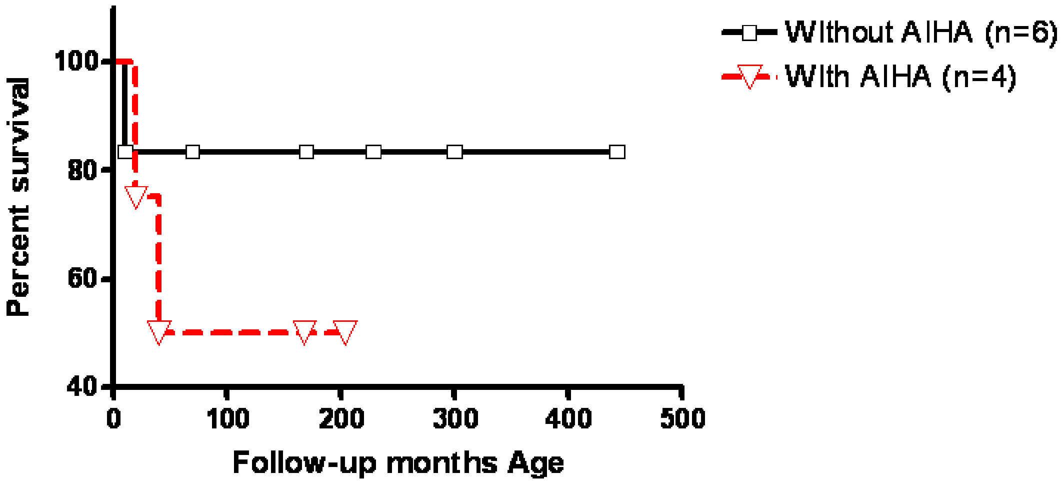

Supplement: Supplementary file 1 [file Data_Sheet_1.pdf]
